# Transitional Care Support for Patients with Heart Failure: A Qualitative Study of Nursing Practices by Ward, Discharge Planning, and Visiting Nurses

**DOI:** 10.24546/0100503457

**Published:** 2026-04-02

**Authors:** MIHO TAKAHASHI, CHIEMI TARU, ATSUKO FUKUDA, IKUKO MIYAWAKI

**Affiliations:** 1 Department of Nursing, Kobe University Graduate School of Health Sciences, Kobe, Japan; 2 Kobe University Hospital, Kobe, Japan; 3 Nonprofit General Incorporated Association, Dweller support net TARUS, Kobe, Japan

**Keywords:** Transitional care, Nursing practices, Heart failure, Collaboration, Content analysis

## Abstract

**AIM:**

This study explores the nursing practices of transitional care support for patients with heart failure performed by three different groups of nurses: 1) ward, 2) discharge planning, and 3) visiting nurses.

**DESIGN:**

We conducted a qualitative, content analysis.

**METHODS:**

Twelve ward nurses, four discharge planning nurses, and five visiting nurses were purposively sampled. Data were collected through semi-structured interviews and analyzed using content analysis.

**RESULTS:**

Ward nurses implemented four practices, including “Encourage patients to improve to prevent re-exacerbations” and “Share information with the home care team.” Discharge planning nurses implemented four practices, including “Communicate with the home care team to ensure the continued management of heart failure.” Visiting nurses implemented three practices, including “Make efforts to obtain test results and, based on physical signs, symptoms, and the pathological condition, detect signs of heart failure exacerbation.”

**CONCLUSION:**

In transitional care support for patients with heart failure, the three different groups of nurses (ward, discharge planning, and visiting) must multilaterally share information on patients’ pathological conditions, the progression of test results, and physical reactions during activities, while continuing to provide support for lifestyle adjustments.

## INTRODUCTION

Heart failure—the fastest-growing cardiovascular disease worldwide—places a tremendous burden on healthcare systems globally (1). The number of patients with heart failure in Japan is gradually increasing as the population ages and is expected to reach 1.3 million by 2030 (2, 3). The readmission rate attributable to heart failure exacerbation is approximately 35% at 2.4 years after discharge (4). To address these issues, the Plan for the Promotion of Measures Against Cerebrovascular and Cardiovascular Disease was formulated in Japan in 2020. This plan calls for the promotion of not only the construction of a comprehensive regional care system that provides medical care, long-term care, and welfare through multidisciplinary collaboration, but also research on cardiovascular disease (5). The prevention of readmissions for chronic diseases is attracting international attention to promote higher quality healthcare and reduce medical costs (6, 7). Nurses play an important role in preparing patients for discharge (8, 9). Effective transitional care is an important nursing task for preventing the acute exacerbation of heart failure and connecting the acute care hospital with the patient’s healthcare and lifestyle after discharge. However, in reality, information regarding nursing practices for adjusting patients’ lives after discharge is not communicated from hospital nurses to visiting nurses (9, 10).

Transitional care is defined as a set of actions designed to ensure the coordination and continuity of healthcare as patients transfer between different locations or different levels of care within the same location (11). The following eight components of effective transitional care have been identified: patient engagement, caregiver engagement, complexity and medication management, patient education, caregiver education, patients’ and caregivers’ well-being, care continuity, and accountability (12).

Several interventions have been studied to reduce the readmission rates of patients with heart failure (6). The following eight themes have been identified as improving long-term outcomes: discharge planning; multidisciplinary collaboration; timely and clear information; medication reconciliation and adherence; engaging social and community support groups; monitoring and managing signs and symptoms after discharge and delivering patient education; outpatient follow-up; and advance care planning and palliative and end-of-life care (6, 13). Furthermore, to ensure continuity of care for patients with heart failure, it is important for nurses acting as the “hub,” to build networks with other healthcare professionals and establish reliable relationships with nursing colleagues (10). In particular, care continuity is an important process for achieving desired care transitions (12); information continuity facilitates management continuity (14, 15). However, nurses perceive hospitals and home health care as two distinct worlds. Even though they share common goals for patients, their nursing practices differ (9). To enhance continuity of care, it is necessary to clarify the specific practices of nurses working in different settings. Nursing practices are intentional (16), meaning they involve acting with purpose and planning with specific goals in mind (17). Therefore, when clarifying nursing practice, it is essential to adopt a perspective that captures not only the content of the practice but also the underlying intent. Furthermore, there is a lack of empirical research on the intentions of nurses working in different settings when providing transitional support for patients with heart failure.

Therefore, this study aimed to clarify the current state of transitional care support for patients with heart failure from the perspective of three groups of nurses: 1) ward nurses in acute care hospitals, 2) discharge planning nurses who specialize in discharge planning, and 3) visiting nurses responsible for home healthcare. By comparing their respective intentional nursing practices, we aim to gain insights into the necessary practices to ensure continuity of care for transitional care support for patients with heart failure.

## MATERIALS AND METHODS

### Definition of Terms

Transitional care support for patients with heart failure, as provided by nurses, refers to nursing practices conducted by ward nurses, discharge planning nurses, and visiting nurses. These nursing practices are intentional (16) and aim to prevent acute exacerbations in patients with heart failure by selecting and coordinating their living arrangements so that the daily lives and healthcare choices of patients discharged from acute care hospitals can be monitored and stabilized.

### Design

Research data were obtained through content analysis, which is well-suited for analyzing the multifaceted and sensitive phenomena characteristic of nursing. The use of inductive content analysis is recommended when knowledge is fragmented, as is the case in this study (18).

### Participants and recruitment

Purposive sampling was employed to obtain information from nurses with extensive experience in heart failure care. We used our connections with facility administrators and visiting nursing specialists on the research team to select our sample. The participants were nurses with at least five years of experience who had assisted patients with heart failure during the transitional period. Five years was chosen because Benner (19) estimated that it takes 3 to 5 years to develop into a mid-career nurse who can view a clinical situation as a whole, rather than from the perspective of a single phase, and focus on the core of the problem. Purposive sampling provided a wealth of information (20). Ward nurses who met the selection criteria and demonstrated a strong interest in heart failure nursing care and transitional care were introduced to this study by the cardiology ward nurse managers. Discharge planning nurses and visiting nurses with extensive experience in transitional care for patients with heart failure were introduced to this study by the co-researcher who has expertise in nursing care for heart failure. After obtaining consent from the nurse managers at each participating facility to conduct the study, the nurses who agreed to participate were included.

### Data collection

Data were collected through semi-structured, in-person interviews with 21 participants from May to October 2019. Information was obtained regarding the interviewees’ sex, age, years of experience as a nurse, years of experience in cardiovascular nursing, years of experience in visiting nursing, years of experience as a certified nurse or certified nurse specialist, and years of experience in discharge planning. Semi-structured interviews were conducted using an interview guide (Table I). The interview guide was developed collaboratively by multiple co-authors, including cardiovascular nursing specialists with expertise in heart failure care, and was it informed by relevant literature (8). Participants were interviewed in a private area of the facilities during or after their shifts. If interviews were conducted during a shift, they were performed with the permission of the nursing manager and in an environment where participants could speak calmly, away from their work.

All interviews were conducted by the first author and recorded on an integrated chip recorder with the participants’ consent. We conducted one interview per participant until no new information emerged.

### Data analysis

Qualitative content analysis (18, 21) was employed. First, we transcribed the interviews individually, carefully read the transcripts repeatedly, and coded all content related to transitional care support for patients with heart failure. We analyzed interview data from each nursing setting. We repeatedly reviewed the verbatim interview data in context. Our focus was on the research question regarding nursing practices that support transitional care for patients with heart failure. Based on similarities and differences, we grouped the data and repeated the coding process to create subcategories and categories. When grouping the data, we considered the intent of nursing practice as revealed by the interview data. For example, we asked, “Why are you concerned about it, what kind of information did you obtain, and how are you applying it to your nursing practice?” Next, to compare nursing practices, we analyzed all subcategories collectively across nurse groups and identified a unifying theme representing the common intentions underlying these practices (22). We performed member checking of interviewee responses immediately during the data collection. When an interviewee’s intentions or the content of their nursing practice were unclear, the lead author confirmed their interpretation of the responses, allowing participants to verify or revise them. MAXQDA 2022 (Ver. 22.4.1) was used to facilitate the data management and coding processes; all coding was performed by the first author. Thereafter, the second author reviewed the coding in detail, and all authors discussed the analysis and results until a consensus was reached.

### Ethical considerations

This study was approved by the Ethics Committee of Kobe University Graduate School of Health Sciences. (approval number: 824-2). Before the interviews, all participants were informed both in writing and verbally about the study’s purpose and methods. Additionally, to avoid coercion from management, we guaranteed that consent or refusal to cooperate in the study would not be reported to nursing managers, that participation in the study would be voluntary, that participants would be free to consent or withdraw their consent, and that anonymity would be strictly maintained. After obtaining the interviewees’ written consent to participate in the study, we arranged a date and time that would not interfere with their work and conducted the interviews at locations where their privacy was protected. Consent for study participation was obtained from all participants.

### Rigor

To ensure reliability and confirmability, we thoroughly described the research setting, participants, methods, and processes (23). The analysis of each nurse in each setting was discussed with a co-researcher who had expertise in nursing care for heart failure and transitional care, as well as experience in qualitative research. We used the Consolidated Criteria for Reporting Qualitative Research (COREQ) (24) as a reference to improve the rigor, comprehensiveness and credibility of the studies. Regarding the analysis, we discussed it with a co-researcher who possesses expertise in heart failure nursing and transitional care, as well as experience in qualitative research. Two members of the research team were experts in qualitative research. Discussions continued until all authors reached consensus.

## RESULTS

Study participants included 21 nurses, including 12 ward nurses with a mean of 13.5 years of experience (range, 6–27 years) and 4 discharge planning nurses with a mean of 24.0 years of experience (range, 12–31 years). There were 5 visiting nurses, with a mean of 26.0 years of experience as a nurse (range, 21–30 years) and a mean of 14.0 years of experience as a visiting nurse (range, 6–20 years). The participants were nurses working in acute care hospitals and visiting nursing stations with 24-hour phone availability in the urban areas of the Kansai region of Japan. Ward nurses were recruited from three participating facilities, discharge planning nurses from four participating facilities, and visiting nurses from three participating facilities. Table II presents the participants’ details.

### Transitional care support for patients with heart failure performed by ward, discharge planning, and visiting nurses

After analyzing the transitional care support for patients with heart failure provided by nurses in each setting, 13 subcategories and 4 categories were extracted for ward nurses (Table III), 14 subcategories and 4 categories for discharge planning nurses (Table IV), and 8 subcategories and 3 categories for visiting nurses (Table V). Owing to the large volume of data, identifiers were assigned to the categories (I–XI) and the subcategories (Ia–XIb). To ensure authenticity, participant data are quoted faithfully in the Appendices and letters in parentheses indicate participant identifiers (A–U).

### Transitional care support for patients with heart failure performed by ward nurses (four categories)

The ward nurses encouraged patients to improve to prevent re-exacerbations (I) and to adjust their amount of activity (II). When preparing the environment for patient recuperation after discharge, they shared information with the discharge planning and outpatient departments (III) as well as the home care team (IV).

### Transitional care support for patients with heart failure performed by discharge planning nurses (four categories)

When discharge planning nurses coordinated a place for the patient to live after discharge (V), they encouraged staff from various professions within the hospital to visualize the patient’s life after discharge (VI) and communicated with the home care team to ensure the continued management of heart failure (VII). Additionally, they maintained a relationship with patients that makes it easy for them to seek advice (VIII).

### Transitional care support for patients with heart failure performed by visiting nurses (three categories)

Instead of highlighting the provision of uniform healthcare guidance for heart failure, visiting nurses emphasized adjusting self-care within the scope of the patient’s desired lifestyle (IX), ensuring that the patient’s life after discharge remains stable. They enable the patient to learn how to engage in activities that avoid cardiac overload (X). Additionally, visiting nurses make efforts to obtain test results and, based on physical signs, symptoms, and the pathological condition, detect signs of heart failure exacerbation (XI).

Next, all subcategories of transitional care support for patients with heart failure were integrated and analyzed to extract seven themes representing the intent of transitional care support for these patients (Table VI).

### The intent of transitional care support for patients with heart failure performed by ward nurses, discharge planning nurses, and visiting nurses (seven themes)

As part of transitional care support, nurses in all settings endeavored to establish and maintain trusting relationships while providing support that respected the wishes of the patient and their family. They also coordinated public and private support systems, striving to prevent the worsening of heart failure. Preventing heart failure exacerbations was emphasized, particularly through medication management and the avoidance of cardiac overload. Support was provided with the intent of continuing heart failure management.

## DISCUSSION

This study examined the current state of transitional care support for patients with heart failure from the perspectives of nurses in various roles. Furthermore, although ward nurses, discharge planning nurses, and visiting nurses involved in transitional care support for patients with heart failure shared the same intentions, their specific nursing practices differed based on the circumstances and the roles of the nurses in each setting. Additionally, variations in transitional care support for patients with heart failure management provided valuable insights into the necessary nursing practices to ensure continuity of care during this phase.

### Setting-specific characteristics and challenges in transitional care support

The ward nurses in this study focused on identifying and supporting areas for improvement to prevent the recurrence of heart failure in transitional care patients. Continuity of care was primarily achieved through information sharing. And when they encouraged patients to adjust the amount of activity, they focused on patients’ balance of activity and rest during hospitalization and provided guidance on how to take a bath, which is generally recommended, as well as preventative measures to avoid experiencing a double burden. In the self-care education of patients with heart failure, ward nurses provide the most education regarding signs and symptoms of worsening conditions and the least education regarding activity (25). Patients with heart failure perceive difficulties in self-management due to a lack of adequate recommendations regarding diet, fluid management, and activities (26). That is, it is important not only to provide general knowledge regarding self-management but also to help patients learn to adjust their lives and manage their symptoms, particularly in their living environments (6). However, a challenge that ward nurses face during transitional care is a lack of time (9). As providing adequate support during a short hospital stay is difficult, the continuity of care for managing learning symptoms should be communicated to the home care team. Visiting nursing has reduced readmissions of patients with heart failure (27). The early introduction of visiting nursing, which assesses pathological conditions, is an important aspect of transitional care support aimed at preventing readmissions.

The specific adjustments made by visiting nurses to avoid cardiac strain—focusing on the patient’s desired lifestyle rather than providing standardized heart failure care guidance—represent essential practices for enhancing self-care abilities and stabilizing the care regimen of patients with heart failure (6). When assessing signs of heart failure exacerbation, they relied on the signs and symptoms exhibited by the body, as well as the underlying pathology, to detect these indicators. However, they made efforts to obtain information about the pathology and test results from the hospital. In Japan, the percentage of pre-discharge conferences conducted at the time of transition to home for patients with heart failure who were newly introduced to the home healthcare system or visiting nursing was reported to be 34.5% (28), which is insufficient. Therefore, to ensure continuity of care, holding a pre-discharge conference is important. Moreover, understanding and assessing the pathophysiology of patients with heart failure is important for maintaining their daily lives after discharge (29, 30). Information sharing between hospital and home physicians regarding disease conditions is lacking (28, 31). Additionally, information sharing and collaboration within home care teams often present challenges, as the organizations they belong to frequently consist of multiple professionals from diverse backgrounds (10)—a situation that also applies in Japan. Therefore, easily obtaining information related to patients’ conditions is challenging for visiting nurses, even when such information is provided by hospital physicians to home physicians. Thus, sharing information about pathological conditions is important for nurses.

The practice of discharge planning nurses as patient advocates—focusing on patient needs, coordinating both within and outside the hospital, and ensuring safe discharge—is consistent with the practices of discharge planning nurses reported in other countries (32, 33). The ward nurses in this study contacted care managers when they collaborated with the home care team, without requesting discharge planning nurses. Care managers are not necessarily medical professionals. It is reported that nurse-led transitional care interventions effectively reduce readmissions in adult patients discharged from acute care hospitals (34). Understanding and evaluating the pathological condition of patients with heart failure to help them maintain their quality of life after discharge is important (29, 30). Cooperating not only with care managers but also with discharge planning nurses and visiting nurses—who are medical professionals—is significant for ward nurses.

### Common intentions and setting-specific practices in transitional care support

The intent of transitional care support for patients with heart failure was consistent among ward nurses, discharge planning nurses, and visiting nurses. However, specific nursing practices differed based on the circumstances and the roles of nurses in each setting. For example, nursing practices intended to coordinate public and private support systems were categorized by ward nurses as practices that encourage patients to improve and prevent re-exacerbations (I), by discharge nurses as practices that coordinate a place to live after discharge (V), and by visiting nurses as practices that adjust self-care within the scope of the patient’s desired lifestyle (IX). These results are supported by previous research that showed that even when there is a common goal in transitional care support, specific practices differ depending on the situation and the nurse’s role (9). It has also been shown that clinical judgment is influenced by the context of the situation and the culture of the nursing care unit (35).

Additionally, seven common themes were identified in this study as intentions for transitional care support for patients with heart failure: establishing and maintaining trust, respecting the wishes of individuals and their families, coordinating public and private support systems, preventing the worsening of heart failure, reliably taking medication, preventing cardiac overload, and continuing with heart failure management. These themes are not contradictory to the eight themes reported to improve long-term outcomes in transitional care for patients with heart failure (6). Therefore, it can be assumed that nurses in each setting were implementing the nursing practices they recognized as necessary in their respective environment, with the common goal of providing seamless transitional care support.

Among these, differences in the current state of transitional care support between ward nurses and visiting nurses—intended to ensure continuity in heart failure management—provided insights into the nursing practices necessary for continuity of care. Specifically, eliminating discrepancies between the information shared by ward nurses during pre-discharge conferences and nursing summaries, and the information visiting nurses require to prevent heart failure exacerbations (such as changes in medical condition and test results, as well as changes in subjective symptoms and vital signs based on activity intensity during hospitalization)—has the potential to promote continuity of nursing care during the transitional care support phase for patients with heart failure.

Comprehensive and effective transitional care requires continuity between inpatient and home care teams (12). Visiting nurses in this study assessed how patients’ conditions affected their daily activities and made detailed lifestyle adjustments. Although the ward nurses in this study indicated that they shared information with the home care team through pre-discharge conferences and nursing summaries, the visiting nurses made efforts to obtain the information they needed. We believe that this is attributable to a lack of knowledge regarding the working environment for visiting nurses, specifically the difficulty in accessing information on pathological conditions, as well as a lack of understanding of the information needed for transitional care support. The discharge planning nurses in this study provided information regarding the patients’ conditions to the home care team to enable the continued management of heart failure. Furthermore, they selected partners based on the severity of the patients’ conditions and the experience of the home care team in managing heart failure, and devised ways to share information. Many of the discharge planning nurses in this study had experience in visiting nursing. Therefore, we believe that they understood the differences in nursing practice across various settings and provided the necessary information for visiting nursing. It is reported that the hospital nurses intended to provide relevant and accurate information; however, their main focus was on fulfilling the standards within the system rather than on the quality of the information exchanged (15). Therefore, the continuity of transitional care support for patients with heart failure may be improved by understanding each other’s working environments and practices, as well as by establishing a method of information sharing that reflects the information necessary for effective practice. Importantly, we gained insights into the detailed information needed by visiting nurses in transitional care support for patients with heart failure. Ward nurses should share this information with visiting nurses during pre-discharge conferences and in nursing summaries to ensure continuity of care.

Respectful and adequate communication, based on a mutual understanding of each other’s positions and roles, results in continuity of care (10, 36). To facilitate transitional care support, it is significant to understand and share each other’s nursing practices and the information necessary for assessment. Especially in transitional care support for patients with heart failure, nurses are required to share information on patients’ pathological conditions, the progression of test results, and physical reactions during activities while continuing to provide support for lifestyle adjustments.

### Strengths and limitations

This study was conducted with experienced discharge planning nurses and visiting nurses. Therefore, the strength of this study lies in the inclusion of certified nurses and certified nurse specialists, as well as the identification of detailed nursing practices. However, certified chronic heart failure nurses who specialize in the care of heart failure patients were not included. Future studies should include certified chronic heart failure nurses to validate the generation of new data. A limitation of this study is that the participants were nurses working in acute care hospitals and visiting nursing stations with 24-hour phone availability in urban areas of the Kansai region of Japan. When assessing the transferability of the results, the differences in regional characteristics and healthcare service systems in other countries should be considered.

## CONCLUSION

This study investigated nursing practices from the perspectives of ward nurses, discharge planning nurses, and visiting nurses involved in transitional care support for patients with heart failure. To facilitate transitional care support, it is important to understand and share each other’s nursing practices and the information necessary for assessment. The current findings imply that it would be useful to standardize and develop an information-sharing tool that includes details of patients’ pathological conditions, the progression of their test results, and their physical reactions during activities, especially in transitional care support for patients with heart failure.

## Figures and Tables

**Table I:** Interview guide

What are your concerns when supporting patients with heart failure in preparation for discharge? Why are you concerned about it? What kind of information was obtained and how does that information relate to their nursing practices? Are you concerned about the results of the inspection? How do you plan to use that information? What kind of information is exchanged during the pre-discharge conference? When the participants did not provide details regarding transitional care support, the following questions were added. What kind of information have you obtained regarding pathology, and how are you applying it to your nursing practice? What kind of information have you obtained regarding the connections that support your patients, and how are you applying it to your nursing practice? What kind of information have you obtained regarding patients’ confidence in their self-management behavior after discharge, and how are you applying it to your nursing practice?

**Table II:** Background of participants

	Nurse	Age (years)	Experience in nursing (years)	Experience in cardiovascular nursing (years)	Experience in visiting nursing (years)	Experience in discharge planning (years)	Certified Nurse/Certified Nurse Specialist Nursing area	City	Number of beds Approx.
Ward nurse	A	40s	25	8	0	0	—	a	600
	B	30s	12	12	0	0	—	a	600
	C	30s	15	10	0	0	—	a	600
	D	30s	13	10	0	0	—	a	600
	E	40s	27	8	0	0	—	a	600
	F	30s	10	10	0	0	—	b	100
	G	30s	11	4	0	0	—	b	100
	H	30s	11	11	0	0	—	c	900
	I	20s	6	5	0	0	—	c	900
	J	30s	11	3	0	0	—	c	900
	K	20s	6	2	0	0	—	c	900
	L	30s	15	7	0	0	—	c	900

Discharge planning nurse	M	30s	12	6	0	6	CNS in Home Care	a	600
	N	50s	22	10	5	10	CN in Visiting Nursing	d	200
	O	50s	31	21	20	1	CN in Visiting Nursing	e	400
	P	50s	31	6	18	5	—	c	900

Visiting nurse	Q	50s	30	15	6	9	—	c	—
	R	40s	28	24	20	0	CN in Visiting Nursing	c	—
	S	40s	22	17	12	0	CN in Visiting Nursing	f	—
	T	50s	29	22	16	0	CN in Visiting Nursing	f	—
	U	40s	21	19	16	0	CN in Dementia Nursing	f	—

CN, Certified Nurse; CNS, Certified Nurse Specialist.

**Table III:** Ward nurse: categories and subcategories: Availability of code applicable to each subcategory for each participant (n = 12)

Category (category no.)	Subcategory (subcategory no.)	Study participant ID											
		A	B	C	D	E	F	G	H	I	J	K	L
Encourage patients to improve to prevent re-exacerbations (I)	Provide guidance focused on prioritizing factors that reduce heart failure exacerbations for the patient (Ia)	●	●	●	●	●	●	●	●	●	●	●	●

	Teach individuals how to self-monitor and recognize that breathing difficulty and tiredness are symptoms of heart failure (Ib)	●	●	●	●	●	●	●	●	●	●	●	●

	Assess medication adherence and implement methods of medication management that address the reasons preventing medication intake (Ic)	●	●	●	●		●	●	●	●	●	●	●

	Respect the wishes of the patient and their family, and collaborate with them and other professionals to resolve differences in self-care (Id)	●	●	●	●	●	●	●	●	●	●	●	●

	Check how the patient is supported by family and suggest the use of long-term care insurance when necessary (Ie)	●	●	●	●	●	●	●	●	●	●		●

	Make a conscious effort to create time to engage with patients (If)	●	●	●	●		●	●				●	●

Encourage patients to adjust the amount of activity (II)	Assess the state of heart failure based on the underlying disease, test results, and subjective symptoms, and balance activity and rest during hospitalization (IIa)	●	●	●	●	●	●	●	●	●	●	●	●

	Advise the patient on what the doctor or physical therapist has confirmed regarding the allowed amount of movement (IIb)		●			●	●	●	●		●	●	●

	Provide guidance on how to take a bath, which is generally recommended as a preventive measure to avoid a double burden (IIc)	●	●	●	●	●	●	●	●	●	●	●	●

Share information with the discharge planning and outpatient departments (III)	If the risk of re-exacerbation is high, share information with the outpatient department (IIIa)						●	●					

	If discharge from the hospital is difficult, request that the discharge planning department arrange for a place of recuperation or to introduce home care services (IIIb)	●	●	●	●	●	●	●	●	●	●	●	●

Share information with the home care team (IV)	Collaborate with the home care team through the care manager (IVa)		●					●	●	●	●		●

	Share information with visiting nurses and care managers at the pre-discharge conference and through nursing summaries (IVb)	●	●	●	●	●	●	●	●	●	●	●	●

**Table IV:** Discharge planning nurse: categories and subcategories: Availability of code applicable to each subcategory for each participant (n = 4)

Category (category no.)	Subcategory (subcategory no.)	Study participant ID			
		M	N	O	P
Coordinate a place to live after discharge (V)	Ask for details regarding daily life and find out how to balance life and self-care (Va)	●	●	●	●

	Comprehensively assess the wishes of the patient and family, the home-based medical care system in the residential area, and the predicted course of the illness to determine the most appropriate place for care (Vb)			●	●

	Check the support capabilities of those around the patient, including financial support, and introduce home care services (Vc)	●	●	●	●

	Based on changes in cardiac function, living environment, and symptoms during activity, arrange a place for recuperation to avoid cardiac overload (Vd)		●	●	●

Encourage staff from various professions within the hospital to visualize the patient’s life after discharge (VI)	Encourage ward nurses to develop nursing practices by visualizing patients’ lives after discharge (VIa)		●	●	●

	Suggest to ward nurses and pharmacists ways to manage medications that patients can continue after their discharge from the hospital (VIb)	●	●	●	●

	Share information on patient care at home within a multidisciplinary team (VIc)		●	●	

Communicate with the home care team to ensure the continued management of heart failure (VII)	Depending on the home care team’s experience in managing heart failure, provide specific explanations to continue the necessary management of heart failure (VIIa)	●	●	●	●

	Depending on the severity, communicate with a home care team experienced in managing heart failure (VIIb)	●		●	●

	Communicate with visiting nurses for the early detection of signs of heart failure exacerbation and request lifestyle adjustments (VIIc)	●	●	●	●

	Act as a point of contact between the home care team and the hospital (VIId)		●	●	●

Maintain a relationship with patients that makes it easy for them to seek advice (VIII)	Thoroughly understand and listen to the patient’s way of thinking and preferences regarding their own lifestyle choices (VIIIa)	●	●	●	●

	Never miss an opportunity when patients request assistance (VIIIb)	●	●	●	

	Establish a relationship that makes it easy for patients to ask for help when they need it (VIIIc)	●	●		

**Table V:** Visiting nurse: categories and subcategories: Availability of code applicable to each subcategory for each participant (n = 5)

Category (category no.)	Subcategory (subcategory no.)	Study participant ID				
		Q	R	S	T	U
Adjust self-care within the scope of the patient’s desired lifestyle (IX)	Create a comfortable environment where patients can live the lives they want (IXa)	●	●	●	●	●

	Determine the specific care needs related to heart failure and provide the necessary self-care (IXb)	●	●	●	●	●

	Identify patient perceptions of their medication and find ways to ensure compliance (IXc)	●	●	●	●	●

	Find a compromise with the patient for viable self-care (IXd)	●	●	●	●	●

Enable the patient to learn how to engage in activities that avoid cardiac overload (X)	Improve patients’ ability to recognize changes in their symptoms during activities (Xa)	●	●	●	●	●

	Identify physical reactions during activities and find ways to avoid cardiac overload (Xb)	●	●	●	●	●

Make efforts to obtain test results and, based on physical signs, symptoms, and the pathological condition, detect signs of heart failure exacerbation (XI)	Detect signs of heart failure exacerbation from the body’s indicators, pathological conditions, and the progression of test results (XIa)	●	●	●	●	●

	Make efforts to hold a pre-discharge conference and obtain test results (XIb)	●	●	●	●	●

**Table VI:** Themes and subcategories

Theme	Nurse	Subcategory (subcategory no.)
Establishing and maintaining trust	WN	Make a conscious effort to create time to engage with patients (If)
	DN	Establish a relationship that makes it easy for patients to ask for help when they need it (VIIIc)
	VN	Create a comfortable environment where patients can live the lives they want (IXa)
Respect for the wishes of the individual and their family	WN	Respect the wishes of the patient and their family, and collaborate with them and other professionals to resolve differences in self-care (Id)
	DN	Comprehensively assess the wishes of the patient and family, the home-based medical care system in the residential area, and the predicted course of the illness to determine the most appropriate place for care (Vb)
	VN	Thoroughly understand and listen to the patient’s way of thinking and preferences regarding their own lifestyle choices (VIIIa)
	VN	Find a compromise with the patient for viable self-care (IXd)
Coordination of public and private support systems	WN	Check how the patient is supported by family and suggest the use of long-term care insurance when necessary (Ie)
	DN	Check the support capabilities of those around the patient, including financial support, and introduce home care services (Vc)
	VN	Determine the specific care needs related to heart failure and provide the necessary self-care (IXb)
Prevention of heart failure worsening	WN	Provide guidance focused on prioritizing factors that reduce heart failure exacerbations for the patient (Ia)
		Teach individuals how to self-monitor and recognize that breathing difficulty and tiredness are symptoms of heart failure (Ib)
	DN	Ask for details regarding daily life and find out how to balance life and self-care (Va)
		Never miss an opportunity when patients request assistance (VIIIb)
	VN	Detect signs of heart failure exacerbation from the body’s indicators, pathological conditions, and the progression of test results (XIa)
Reliable taking medication	WN	Assess medication adherence and implement methods of medication management that address the reasons preventing medication intake (Ic)
	DN	Suggest to ward nurses and pharmacists ways to manage medications that patients can continue after their discharge from the hospital (VIb)
	VN	Identify patient perceptions of their medication and find ways to ensure compliance (IXc)
Prevent cardiac overload	WN	Assess the state of heart failure based on the underlying disease, test results, and subjective symptoms, and balance activity and rest during hospitalization (IIa)
		Advise the patient on what the doctor or physical therapist has confirmed regarding the allowed amount of movement (IIb)
		Provide guidance on how to take a bath, which is generally recommended as a preventive measure to avoid a double burden (IIc)
	DN	Based on changes in cardiac function, living environment, and symptoms during activity, arrange a place for recuperation to avoid cardiac overload (Vd)
	VN	Improve patients’ ability to recognize changes in their symptoms during activities (Xa)
		Identify physical reactions during activities and find ways to avoid cardiac overload (Xb)
Continue with heart failure management	WN	If the risk of re-exacerbation is high, share information with the outpatient department (IIIa)
		If discharge from the hospital is difficult, request that the discharge planning department arrange for a place of recuperation or to introduce home care services (IIIb)
		Collaborate with the home care team through the care manager (IVa)
		Share information with visiting nurses and care managers at the pre-discharge conference and through nursing summaries (IVb)
	DN	Encourage ward nurses to develop nursing practices by visualizing patients’ lives after discharge (VIa)
		Share information on patient care at home within a multidisciplinary team (VIc)
		Depending on the home care team’s experience in managing heart failure, provide specific explanations to continue the necessary management of heart failure (VIIa)
		Depending on the severity, communicate with a home care team experienced in managing heart failure (VIIb)
		Communicate with visiting nurses for the early detection of signs of heart failure exacerbation and request lifestyle adjustments (VIIc)
		Act as a point of contact between the home care team and the hospital (VIId)
	VN	Make efforts to hold a pre-discharge conference and obtain test results (XIb)

WN, Ward nurse; DN, Discharge Planning Nurse; VN, Visiting Nurse.

**Appendix 1 tVII-kobej-71-e154:** Ward nurse

Category (category no.)	Subcategory (subcategory no.)	*Representative data ( participant identifiers)*
Encourage patients to improve to prevent re-exacerbations (I)	Provide guidance focused on prioritizing factors that reduce heart failure exacerbations for the patient (Ia)	*To prevent readmission, nurses need to collect a large volume of information, analyze it, and provide guidance focused on reducing factors that exacerbate heart failure that should be prioritized for the patient (E)*.
	Teach individuals how to self-monitor and recognize that breathing difficulty and tiredness are symptoms of heart failure (Ib)	*Symptoms of heart failure vary from person to person. Some people have difficulty breathing, some have trouble eating, and others feel that their legs are heavy. I ask them to tell me what they noticed that led them to see the doctor, and the next time they have another sign of this, I tell them to see the doctor as soon as possible (H)*.
	Assess medication adherence and implement methods of medication management that address the reasons preventing medication intake (Ic)	*Many patients who can take their medication in the hospital may not be able to do so when they return home. So, if it seems to be difficult for them to manage their medication, we propose using a medication calendar from the early stage of hospitalization (F)*.
	Respect the wishes of the patient and their family, and collaborate with them and other professionals to resolve differences in self-care (Id)	*Personally, I do not like restrictions and want to focus on QOL if they are going to go home. I think about the person’s happiness (H)*.
	Check how the patient is supported by family and suggest the use of long-term care insurance when necessary (Ie)	*If it is unknown whether long-term care will be certified, but the patient’s ADL is impaired, it should be initially proposed that the patient apply for certification and have receive the assessment (G)*.
	Make a conscious effort to create time to engage with patients (If)	*Rather than asking the patient how they feel, I should ask them for details, such as whether their legs are swollen or whether they are eating, so that they can understand what they need to be careful about (L)*.
Encourage patients to adjust the amount of activity (II)	Assess the state of heart failure based on the underlying disease, test results, and subjective symptoms, and balance activity and rest during hospitalization (IIa)	*I tell patients that if the X-ray results get better, they can try moving, or if they get worse, it might be easier to keep their heads elevated (I)*. *I am most concerned about breathing difficulties. The only support I can offer is to tell them that they should take a rest if they are tired (H)*.
	Advise the patient on what the doctor or physical therapist has confirmed regarding the allowed amount of movement (IIb)	*For example, if I provide the physical therapist with information that a patient walks with heavy items when shopping, and the physical therapist says that the patient should have a cart, I give that information to the patient. I am so busy with work that I cannot take time to interact fully with the patient (E)*. *For example, if the patient is a woman, I ask their doctor how much they are allowed to move when doing household chores; if the patient is a man, I ask their doctor how much they are allowed to move when working. Then, I tell the patient to avoid cardiac overload (F)*.
	Provide guidance on how to take a bath, which is generally recommended as a preventive measure to avoid a double burden (IIc)	*As every day (showering) is a cardiac overload for the patient, I give general explanations to avoid a double burden (E)*. *Generally, we instruct them to take a bath in lukewarm water or soak only the lower half of their body in warm water, but we have not been able to give them personalized care instructions (F)*.
Share information with the discharge planning and outpatient departments (III)	If the risk of re-exacerbation is high, share information with the outpatient department (IIIa)	*We assess the patients who have insufficient self-management or who are at high risk of readmission while they are staying in the hospital. They are then supported by nutritional guidance at outpatient clinics and by outpatient nursing (F)*.
	If discharge from the hospital is difficult, request that the discharge planning department arrange for a place of recuperation or to introduce home care services (IIIb)	*For patients who have a visiting nursing or care manager, the discharge planning department coordinates with them. If it seems difficult to discharge to home, we consult with the discharge planning department to discuss and decide the best way to do so (C)*. *I want to introduce visiting nurses to patients with heart failure, but since they look healthy, I hear that visiting nursing service is not possible for those with long-term care insurance. I heard that visiting nursing care is available with medical insurance, but my knowledge is limited, so I consult with the social workers (K)*.
Share information with the home care team (IV)	Collaborate with the home care team through the care manager (IVa)	*Patients with little family support usually have a care manager, so I communicate with that person (B)*. *I only know what happens during hospitalization, so I have to ask the care manager about the progress up to the time of hospitalization (L)*.
	Share information with visiting nurses and care managers at the pre-discharge conference and through nursing summaries (IVb)	*Inform the staff (at the pre-discharge conference) of blood pressure changes, fluid restriction, weight goals, medications, ADLs, family environment, and the patient’s perception of their illness (D)*. *I believe that we are informative in our nursing summaries (E)*.

**Appendix 2 tVIII-kobej-71-e154:** Discharge planning nurse

Category (category no.)	Subcategory (subcategory no.)	*Representative data ( participant identifiers)*
Coordinate a place to live after discharge (V)	Ask for details regarding daily life and find out how to balance life and self-care (Va)	*From the time they wake up until they go to bed, we ask them about their daily lives in detail, such as how they take a bath, what they eat, and who does the shopping. We first show what is possible with the patient’s cardiac function, such as hydration, salt intake, and exercise, and then discuss details together with the patient and family (N)*.
	Comprehensively assess the wishes of the patient and family, the home-based medical care system in the residential area, and the predicted course of the illness to determine the most appropriate place for care (Vb)	*If the patient and family have a clear intention, discharge to home is possible, though it may be difficult at times because it is affected by the medical situation in the area where the patient is returning (P)*. *We predict the prognosis based on doctor’s records, cardiac function, and disease, and if we find it difficult to discharge to home, we make adjustments at the same time as coordinating the facility to live and apply for long-term care insurance (O)*
	Check the support capabilities of those around the patient, including financial support, and introduce home care services (Vc)	*If their family members are unable to support them in their daily lives, they are worried, but I try to provide them with home care services to help them feel reassured (M)*. *Financial circumstances also have a large influence on the decision of whether to use visiting nursing (P)*.
	Based on changes in cardiac function, living environment, and symptoms during activity, arrange a place for recuperation to avoid cardiac overload (Vd)	*First, we check based on the patient’s condition to see if it is possible to do what they want to do after being discharged from the hospital. We help them bridge the gap between what they want to do and their cardiac function (N)*. *If the patient has become accustomed to going shopping even if there are hills or stairs, it cannot be corrected unless the patient is told that this is overactivity (O)*.
Encourage staff from various professions within the hospital to visualize the patient’s life after discharge (VI)	Encourage ward nurses to develop nursing practices by visualizing patients’ lives after discharge (VIa)	*Ward nurses tend to miss the assessment of the living environment and cleanliness. I tell them that the patient lives on the third story with many slopes around them and ask them to check the load on slopes and stairs and the SpO_2_ in the shower (O)*.
	Suggest to ward nurses and pharmacists ways to manage medications that patients can continue after their discharge from the hospital (VIb)	*Ask the pharmacist if it is possible for the patient to take home the medication calendar they have been using in the hospital (O)*. *When the hospital uses boxes and calendars of different colors and shapes to manage medication, patients will not be able to take their medication after being discharged from the hospital. I tell the ward nurses to think of ways to help patients take their medications at home (P)*.
	Share information on patient care at home within a multidisciplinary team (VIc)	*I tell the dietitians the stores where the patients go shopping and the types of foods they buy; I also tell the dietitians that I want them to take this into account and give the patients dietary guidance (O)*. *A multidisciplinary team taking care of patients with heart failure gathers to integrate opinions and unify the direction of support to give (N)*.
Communicate with the home care team to ensure the continued management of heart failure (VII)	Depending on the home care team’s experience in managing heart failure, provide specific explanations to continue the necessary management of heart failure (VIIa)	*Some visiting nursing stations and facilities may not be aware of the symptoms of heart failure, so we give detailed information on what to watch out for (O)*. *I tell the care manager how much exertion can cause the patient to become short of breath, how well the patient’s cardiac function is, and how well the patient’s actual movement is. This is the kind of support that is needed (P)*.
	Depending on the severity, communicate with a home care team experienced in managing heart failure (VIIb)	*In cases of severe heart failure, I will communicate with a competent visiting nurse station in cardiovascular care (O)*.
	Communicate with visiting nurses for the early detection of signs of heart failure exacerbation and request lifestyle adjustments (VIIc)	*Nurses are important because they can assess life and heart failure and detect signs of exacerbation. In cases wherein readmission is required immediately, it is important to have a daily life assessment nearby, so we arrange visiting nurses (P)*.
	Act as a point of contact between the home care team and the hospital (VIId)	*We attend the visiting nursing management meetings and establish a mutual acquaintance with each other. Occasionally, a support person at home calls the hospital when they have a problem about a patient, and I contact the doctor to see the patient (N)*.
Maintain a relationship with patients that makes it easy for them to seek advice (VIII)	Thoroughly understand and listen to the patient’s way of thinking and preferences regarding their own lifestyle choices (VIIIa)	*When a patient’s way of thinking or what they value is slightly different from our values, their life history may be relevant, so we understand this before engaging with the issue (N)*.
	Never miss an opportunity when patients request assistance (VIIIb)	*I try to intervene when the person is in trouble when the SOS from the person comes (M)*.
	Establish a relationship that makes it easy for patients to ask for help when they need it (VIIIc)	*I see them during their outpatient visits, listen to them, and tell them to remember my face when they need me (N)*.

**Appendix 3 tIX-kobej-71-e154:** Visiting nurse

Category (category no.)	Subcategory (subcategory no.)	*Representative data ( participant identifiers)*
Adjust self-care within the scope of the patient’s desired lifestyle (IX)	Create a comfortable environment where patients can live the lives they want (IXa)	*I listen to what the patient really values, and I try to value the same (U)*.
	Determine the specific care needs related to heart failure and provide the necessary self-care (IXb)	*Older adults cannot eat when they are not feeling well, so I do not strongly advise restricting their diet (R)*.
	Identify patient perceptions of their medication and find ways to ensure compliance (IXc)	*As I think there are reasons for not taking the medicine, I try relating to them, so that I hear what is really happening (S)*.
	Find a compromise with the patient for viable self-care (IXd)	*It is impossible for hospital nurses to directly observe patients’ lives. There are some things we can suggest because we go inside the house. I think our strength as visiting nurses is that we can make compromises and communicate details as we talk (U)*.
Enable the patient to learn how to engage in activities that avoid cardiac overload (X)	Improve patients’ ability to recognize changes in their symptoms during activities (Xa)	*Even if shortness of breath lasts for 20 to 30 minutes after taking a bath, the patient does not perceive it as a problem when it becomes normal. If we do not tell them that they are having a huge double burden, we will not be able to provide support (Q)*. *I suggest a comfortable posture. Once you know which postures are uncomfortable, you can change your behavior. However, if you do not know the reason, you cannot improve it. Thus, I repeat the same thing every time I visit a patient so that they can realize it on their own (R)*.
	Identify physical reactions during activities and find ways to avoid cardiac overload (Xb)	*I see differences in the patients’ pulses in the flow lines in the houses after they use the bathroom. I take into consideration the changes in their bodies before and after the movement and their perceived tiredness and suggest ways of movement that do not put stress on the heart (R)*. *I ask patients in detail whether they are taking a bath that supports their elbows, which reduces the burden on the heart, and whether they are taking a hot bath (T)*.
Make efforts to obtain test results and, based on physical signs, symptoms, and the pathological condition, detect signs of heart failure exacerbation (XI)	Detect signs of heart failure exacerbation from the body’s indicators, pathological conditions, and the progression of test results (XIa)	*We want information on the condition of the patient during hospitalization, as we ascertain the heart condition from the heartbeat, respiratory sounds, pulse, and changes in blood pressure. I want to see how bad the symptoms were at the time of admission and how much they improved at the time of discharge. Blood data, echocardiograms, and electrocardiograms show how bad the symptoms were and how much they have improved. When we can predict the response to diuretics, it will be an indicator of how long it is okay to continue using diuretics alone even after discharge from the hospital (R)*.
	Make efforts to hold a pre-discharge conference and obtain test results (XIb)	*Occasionally, there are no pre-discharge conferences or nursing summaries, and certainly, patient referral documents are not readily available. There is very little medical information from care managers, so it is important to properly share information among medical professionals. It is nearly impossible for visiting nurses to know the course of test results (Q)*. *At the pre-discharge conference, we ask the physical therapist about changes in vital signs and SpO_2_ during exercise, how big are the patient’s strides when walking in the ward, and at what heart rate they need to rest (T)*.
